# RNA-expression of adrenomedullin is increased in patients with severe COVID-19

**DOI:** 10.1186/s13054-020-03246-1

**Published:** 2020-08-28

**Authors:** Julian Hupf, Julian Mustroph, Frank Hanses, Katja Evert, Lars S. Maier, Carsten G. Jungbauer

**Affiliations:** 1 Emergency Department, University Hospital Regensburg, Franz-Josef-Strauß-Allee 11, 93053 Regensburg, Germany; 2 Department of Internal Medicine II (Cardiology), University Hospital Regensburg, Franz-Josef-Strauß-Allee 11, 93053 Regensburg, Germany; 3 Department of Infection Prevention and Infectious Diseases, University Hospital Regensburg, Franz-Josef-Strauß-Allee 11, 93053 Regensburg, Germany; 4 Institute of Pathology, University of Regensburg, Franz-Josef-Strauß-Allee 11, 93053 Regensburg, Germany

Adrenomedullin (ADM) is a peptide hormone with vasodilatory effects and involved in the regulation of the endothelial barrier function. Previous research found increased ADM plasma levels in patients with sepsis and ADM levels correlated with disease severity and mortality in sepsis [1]. Although severe coronavirus disease (COVID-19) shares some clinical features of sepsis (e.g., endothelial barrier dysfunction [2]), it is not known whether pathophysiological pathways of COVID-19 resemble those of sepsis [2, 3]. To our knowledge, this is the first study to evaluate ADM in context of COVID-19 [4].

We present here data regarding ADM in patients with COVID-19. Starting from March 2020, we included 45 adult patients presenting with signs of respiratory infection (cough and/or fever) to the Emergency Department in this ongoing study. The study was approved by the ethics committee of the University of Regensburg. Each individual provided written informed consent prior to inclusion. SARS-CoV-2 infection status was evaluated by PCR analysis mainly using throat rinse water (or less frequently nasopharyngeal swabs). Patients were classified as COVID-19 positive (PCR positive for SARS-CoV-2 and signs of respiratory infection) or control (other viral or bacterial respiratory infection). Whole blood was drawn by venipuncture and lysed in Trifast (Ambion) buffer solution. Further, RNA expression analysis of ADM in whole blood was performed using qPCR and normalized to GAPDH as housekeeper gene. The final diagnosis after patient discharge was reviewed by a consultant physician and patients without evidence of respiratory infection were excluded from analysis (*n* = 5).

Baseline characteristics of the study population are described in Table 1. The risk of clinical deterioration estimated by NEWS-2 Score [5] did not differ between both groups. Six patients in the COVID-19 group were admitted to the ICU (defined as “severe COVID-19”), four of them required mechanical ventilation during hospital stay and three of them died due to COVID-19 or related complications. In contrast, only one patient in the control group died from pneumonia.

**Table 1:** Baseline characteristics of the study population

	COVID-19	Controls	
*n*	21	19	
Sex male	57.1%	57.9%	*p* = n.s.*
Age^a^	50 ± 16	56 ± 20	*p* = n.s.#
ICU admission	28.6%	5.3%	*p* = n.s.*
Death	19.0%	5.3%	*p* = n.s.*
Coronary artery disease	4.8%	36.8%	*p* = 0.02*
Chronic heart failure	4.8%	15.8%	*p* = n.s.*
Chronic obstructive pulmonary disease	0.0%	21.1%	*p* = 0.04*
Hypertension	28.6%	47.4%	*p* = n.s.*
Hyperlipidemia	9.5%	36.8%	*p* = n.s.*
Diabetes	4.8%	26.3%	*p* = n.s.*
Chronic renal failure	9.5%	21.1%	*p* = n.s.*
Heart frequency [bpm]^a^	96 ± 15	92 ± 27	*p* = n.s.#
Systolic blood pressure [mmHg]^a^	132 ± 20	134 ± 28	*p* = n.s.#
Oxygen saturation^a^	95 ± 4%	95 ± 3%	*p* = n.s.#
Respiratory rate [per minute]^a^	22 ± 7	21 ± 7	*p* = n.s.#
Temperature [°C]^a^	37.7 ± 0.7	37.4 ± 0.9	*p* = n.s.#
BMI^a^	27.8 ± 5.6	27.6 ± 5.3	*p* = n.s.#
NEWS-2 Score^b^	4 ± 5	3 ± 4	*p* = n.s.#
Creatinine [mg/dl]^b^	0.88 ± 0.58	0.95 ± 0.55	*p* = n.s.#
CRP [mg/l]^b^	43.4 ± 66.7	48.4 ± 74.0	*p* = n.s.#
WBC [/nl]^b^	7.2 ± 4.7	8.1 ± 7.9	*p* = n.s.#
IL-6 [pg/ml]^b^	30.7 ± 50.1	35.8 ± 87.5	*p* = n.s.#
ADM/GAPDH expression^a^	0.88 ± 0.45	0.58 ± 0.35	*p* = 0.025

^*^Tested with Fisher’s exact test

^#^Tested with Mann-Whitney *U* test

^a^Values are mean ± standard deviation

^b^Values are median ± interquartile range

ADM expression was significantly elevated in patients with COVID-19 than other respiratory infections (Fig. 1a) despite similar clinical features at admission. In patients with COVID-19, ADM expression was significantly higher in patients with severe COVID-19 than in patients with less severe COVID-19 (Fig. 1b). Further, ADM expression was not significantly different between patients with less severe COVID-19 and patients with other respiratory infections than COVID-19 (*p* = n.s.). According to ROC-analysis, ADM was able to differentiate severe from non-severe COVID-19 cases with an AUC of 0.82 (*p* = 0.024, 95% CI 0.64–1.0).

**Fig. 1:**
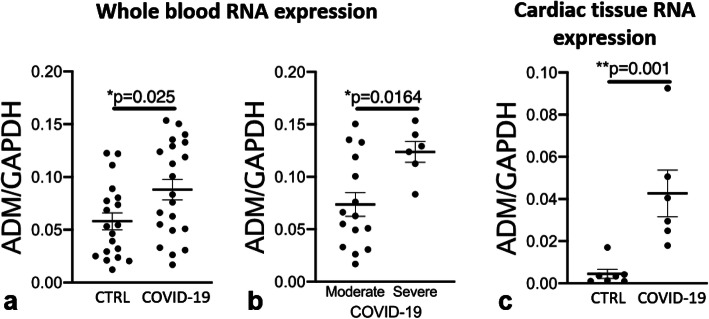
Adrenomedullin RNA expression in whole blood is significantly increased in patients with COVID-19 versus other respiratory infections (“CTRL”) in whole blood (**a**, Student’s *t* test). Further, ADM is significantly elevated in patients with severe COVID-19 in contrast to moderate disease (**b**, Student’s *t* test). ADM expression in myocardial tissue is increased in patients, who died from COVID-19, in comparison to controls (“CTRL”, **c**, Mann-Whitney U test). Values are depicted as mean and standard error of the mean

To strengthen our hypothesis, we analyzed ADM expression in the left ventricular myocardial tissue of patients who were deceased from COVID-19. Infection with SARS-CoV-2 had been verified by PCR in all of these patients. As control, we used a combination of left ventricular myocardial tissue of patients who died from other respiratory infections or from patients destined for organ donation, which could ultimately not be performed. We found a significantly elevated expression of ADM in patients who died from COVID-19 in contrast to controls (Fig. 1c).

Our findings suggest a potential role for ADM in severe COVID-19. While ADM might be a therapeutic target in sepsis and septic shock, further research is needed regarding ADM in COVID-19. Further, the diagnostic potential of ADM as a marker for progression to severe COVID-19 at first medical contact should be evaluated.

Limitations of this study are the small number of patients included and RNA expression analysis in contrast to direct measurement of ADM levels. This study, however, is the first to show an association between ADM and severity of COVID-19.

## Data Availability

The datasets used and/or analyzed during the current study are available from the corresponding author on reasonable request.
